# Fractional flow reserve versus intravascular ultrasound during percutaneous intervention

**DOI:** 10.1097/MS9.0000000000002313

**Published:** 2024-06-26

**Authors:** Hasaan Ahmed, Mahmoud Ismayl, Anirudh Palicherla, Ruth Kalathil, Christian Patterson, Suma Pusapati, Terezia Petraskova, Jalal Dufani, Amjad Kabach, Ahmed Aboeata

**Affiliations:** a Department of Medicine, Division of Internal Medicine, Creighton University School of Medicine, Omaha; b Department of Medicine, Division of Cardiovascular Disease, Creighton University School of Medicine, Omaha, NE; c Department of Cardiovascular Medicine, Mayo Clinic, Rochester, MN, USA

Coronary angiography remains the gold standard for assessing the severity of coronary artery disease during percutaneous coronary intervention (PCI)^1^. Prognostic indications of coronary artery disease are multifaceted, encompassing plaque burden, luminal narrowing, and physiological dynamics^2^. While fractional flow reserve (FFR) has been the conventional adjunctive modality for assessing the physiological significance of coronary lesions, intracoronary imaging with intravascular ultrasound (IVUS) has become increasingly utilized^1–3^. Despite the prevalence of both modalities in PCI, clinical outcomes between modalities remain unclear. Therefore, we performed a meta-analysis to evaluate the clinical outcomes of FFR versus IVUS among patients undergoing PCI.

A comprehensive literature search was performed utilizing PubMed, EMBASE, ClinicalTrials.gov, and Cochrane databases for studies evaluating outcomes of FFR versus IVUS among patients undergoing PCI. The following search terms/keywords were used both individually and in combination: ‘Percutaneous coronary intervention,’ ‘Fractional flow reserve,’ ‘intravascular ultrasound,’ and ‘Coronary lesions.’ Two evaluators (H.A. and M.I.) used a two-step screening process for title, abstract, and full-text screening. Any disagreements that arose among reviewers were addressed through extensive discussions. Outcomes of interest were all-cause mortality and target vessel revascularization. A common-effect model was used to calculate risk ratios (RR) with 95% confidence intervals (CI) in R studio, version 2024.07, for each outcome assessed. Heterogeneity was assessed using the *I* ^2^ test.

A total of three studies were identified^1–3^. The total study population encompassed 2013 patients, of which 995 underwent FFR-guided PCI, and 1018 underwent IVUS-guided PCI. The patient population included those with stable coronary artery disease and acute coronary syndrome. Two studies compared the efficacy of IVUS-guided PCI versus FFR-guided PCI in intermediate coronary lesions (40–70% stenosis), and one study compared the efficacy of IVUS-guided PCI versus FFR-guided PCI in hemodynamically significant long lesions (FFR≤0.80). Among those with intermediate coronary lesions, 921 patients underwent FFR-guided PCI, and 938 patients underwent IVUS-guided PCI. Among those with hemodynamically significant long lesions, 74 patients underwent FFR-guided PCI, and 80 patients underwent IVUS-guided PCI. There were no significant differences between FFR-guided PCI versus IVUS-guided PCI in all-cause mortality (RR 0.59; 95% CI: 0.30–1.18, Fig. 1A) and target vessel revascularization (RR 1.48; 95% CI: 0.90–2.44, Fig. 1B).

**Figure 1:**
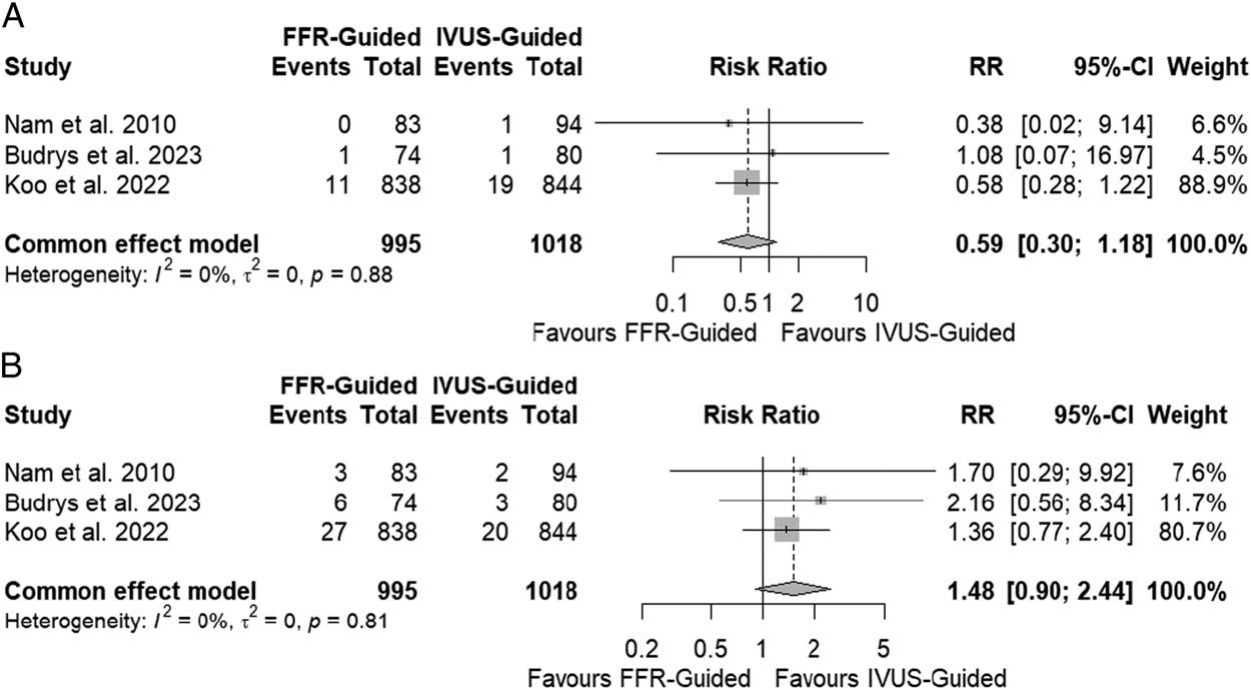
Forest plots of outcomes assessed. (A) All-cause mortality. (B) Target vessel revascularization.

Our results suggest that it is reasonable to proceed with IVUS or FFR among patients undergoing PCI. A review of the literature suggests enhanced efficacy with physiological evaluation in ischemia-targeted PCI, whereas intracoronary imaging excels in the preparation process of PCI and in evaluating anatomical features^2^. Although IVUS and FFR were created to address two different aims, operators often use these methods interchangeably^2^. Given the recognition of ischemia, plaque characteristics, and PCI appropriateness as crucial prognostic indicators, our results highlight the need to expand upon the effectiveness of physiological versus intracoronary imaging-mediated modalities in PCI^2^.

Our findings warrant discussion of existing literature regarding comparative interventions among patients with coronary artery disease. In a meta-analysis evaluating outcomes of polymer-free versus polymer-coated drug-eluting stents by Khatri and colleagues, there was a mildly reduced risk of all-cause mortality with the use of polymer-free drug-eluting stents (*P*=0.05), while outcomes of cardiovascular mortality and noncardiovascular mortality were similar between both types of stents, respectively^4^. Furthermore, male sex and prior myocardial infarction were noted to be independently associated with an increased risk of all-cause mortality and cardiovascular mortality^4^. Although our findings noted no significant differences between FFR-guided PCI and IVUS-guided PCI in all-cause mortality, further studies are warranted to assess the impact of sex on adverse outcomes among patients undergoing PCI with adjunctive modalities.

Our findings of IVUS being noninferior to FFR in all-cause mortality and target vessel revascularization reflect the limitations of coronary angiography, marked by its two-dimensional imaging and incapacity to fully assess internal arterial dimensions and plaque composition, all of which have spurred the development of intracoronary imaging technologies^5,6^. The implementation of these adjunctive imaging modalities has augmented angiographic data by providing enhanced accuracy of vessel dimension measurements, improved lesion characterization to facilitate optimal vessel preparation, and refined stent sizing^7,8^. Evidently, enhanced clinical outcomes have been well-recognized in prior literature with the use of intracoronary imaging, propagated by reductions in adverse cardiac events, target‐lesion revascularization, and target‐vessel failure^9^.

Our study has several limitations. Due to the limited number of studies in our analysis, we could not perform a regression analysis, thereby limiting our ability to adjust for confounders.

Although this study was primarily focused on adjunctive modalities with PCI, we acknowledge that other factors may also adversely affect outcomes such as operator experience, facility capabilities, sex, race, and socioeconomic disparities^6^. Additionally, not all of the included studies in our meta-analysis specifically differentiated between coronary lesion types or classifications based on the Society for Coronary Angiography and Interventions (SCAI) guidelines. While our study was unable to stratify our study population based on these factors, future studies would benefit from exploring the impact of coronary lesion characteristics and SCAI classification on outcomes and procedural success rates with the use of adjunctive modalities during PCI.

In conclusion, our findings suggest that IVUS-guided PCI was noninferior to FFR-guided PCI in all-cause mortality and target vessel revascularization. Our study may serve as a framework for procedural considerations among patients undergoing PCI. Further studies are needed to confirm our findings.

## Ethical approval

Ethical approval was not required for this editorial.

## Consent

Informed consent was not required for this editorial.

## Source of funding

This research did not receive any specific grant from funding agencies in the public, commercial, or not-for-profit sectors.

## Author contribution

H.A., M.I., A.P., R.K., C.P., S.P., T.P., J.D., A.K., A.A: conceptualization; H.A., M.I., A.P., R.K., C.P., S.P., T.P.: writing – original draft; H.A., M.I., A.P., R.K., C.P., S.P., T.P., J.D., A.K., A.A.: writing – review and editing; H.A., M.I., A.P.: investigation; J.D., A.K., A.A: supervision.

## Conflicts of interest disclosure

The authors report no conflicts of interest.

## Research registration unique identifying number (UIN)

Not applicable.

## Guarantor

Hasaan Ahmed, MD.

## Data availability statement

The data which supports the findings of this manuscript are openly available upon reasonable request from the corresponding author.

## Provenance and peer review

Not commissioned, externally peer-reviewed.
